# Evidence of obesity-induced inflammatory changes in client-owned cats

**DOI:** 10.14202/vetworld.2024.1685-1692

**Published:** 2024-08-04

**Authors:** Steffi L. Araujo, Patricia L. Martins, Thyago H. de Souza Pereira, Tiago L. Sampaio, Ramon R. Paula Pessoa Bezerra de Menezes, Mac D. Rodrigues da Costa, Alice M. Costa Martins, Isaac Neto Goes da Silva, Glayciane Bezerra de Morais, Janaina Serra Azul Monteiro Evangelista

**Affiliations:** 1 Laboratory of Comparative Experimental Morphology, Faculty of Veterinary, State University of Ceará, Fortaleza, 60714-903, Ceará, Brazil; 2 Animal Physiology Laboratory, Federal Rural University of the Amazon, Pará, 66077-830, Belém, Brazil; 3 Department of Clinical and Toxicological Analysis, Faculty of Pharmacy, Dentistry and Nursing, Federal University of Ceará, Fortaleza, 60430-275, Ceará, Brazil; 4 Laboratoy of Veterinary Clinical Pathology, Faculty of Veterinary, State University of Ceará, Fortaleza, 60714-903, Ceará, Brazil

**Keywords:** adipokines, cytokines, feline, obese

## Abstract

**Background and Aim:**

Insulin resistance and type 2 diabetes mellitus are common health issues in obese (OB) cats. In humans, obesity leads to alterations in adipokine and proinflammatory cytokine secretion, causing persistent inflammation. The inflammatory impact of obesity in cats remains unproven. This study investigated associations between obesity and inflammatory and metabolic changes in three groups of client-owned Brazilian domestic shorthair cats: naturally lean, overweight (OW), and OB.

**Materials and Methods:**

Cats from the Veterinary Hospital of Professor Sylvio Barbosa e Cardoso (FAVET/UECE) were clinically evaluated. Blood samples were collected for hematological and biochemical profile measurements, and part of the serum was used for measuring adipokine and inflammatory cytokines using sandwich enzyme-linked immunosorbent assay.

**Results:**

In both the OW and OB groups, serum cholesterol and insulin concentrations increased, while triglyceride concentrations were notably elevated in the OB group. In the OW and OB groups, serum adiponectin, tumor necrosis factor-α, and interleukin-1β levels were elevated, and leptin levels were significantly higher in the OB group.

**Conclusion:**

This study is the first in Brazil to reveal increased serum levels of inflammatory markers in OW and OB client-owned felines. OW cats exhibited higher proinflammatory marker levels, implying obesity-induced inflammation.

## Introduction

Obesity, a common endocrine affliction in cats [1], is acknowledged to be a considerable animal welfare concern [2, 3] due to its heightened risk of mortality and shortened lifespan [4, 5]. This issue, which arises from genetic and environmental factors [6, 7], is linked to numerous diseases such as diabetes mellitus [8], hepatic lipidosis [9], and a spectrum of orthopedic [10], respiratory [11], and urogenital disorders [12, 13]. The resemblance between feline diabetes mellitus and human type 2 diabetes [14] is strengthened by the finding that overweight (OW) cats suffer decreased insulin sensitivity [15].

Adipokines such as leptin and adiponectin, secreted by adipose tissue, regulate various metabolic functions. In the face of nutritional overload, excessive fat accumulation in adipocytes appears to cause cellular stress and dysfunction, which may lead to inflammatory responses in the adipose tissue [16]. Adipocytes with hypertrophy can induce an inflammatory reaction in adipose tissue through the release of excess adipokines and cytokines [17]. Macrophages and adipocytes interact, amplifying inflammatory signals in adipose tissue, thereby promoting the development of “low-grade” chronic inflammation in obesity [18, 19]. The release of tumor necrosis factor (TNF)-α, interleukin (IL)-1, and IL-6, proinflammatory cytokines, and chemokines is heightened in obesity-related inflammation [20].

Despite the physiological similarities in the mechanisms involved in obesity between humans and cats, studies proving the inflammatory state in obese (OB) cats are scarce. Obesity-related changes in adipokine and cytokine production have been identified in feline studies, but these studies do not all agree, and their findings may be due to the limited number of cats included [21–23]. This study investigated the associations between obesity and inflammatory and metabolic changes in naturally lean, OW, and OB, neutered, client-owned Brazilian domestic shorthair cats.

## Materials and Methods

### Ethical approval and informed consent

The study was approved by the Ethics Committee for the use of animals at the Universidade Estadual do Ceará (protocol number 23062020/2020). The owners of the cats expressed consent to participate in the study by signing an official document.

### Study period and location

This study was conducted from May 2021 to July 2023. All cats were obtained from the Veterinary Hospital of the State University of Ceará, Fortaleza, Ceará, Brazil.

### Animals

This prospective study analyzed 51 Brazilian domestic shorthair cats (*Felis catus*) of both sexes, aged between 1 and 7 years. The enrolled cats were divided into three groups: OW group, 19 cats with body condition scoring (BCS) 6–7/9; OB group, 16 cats with BCS 8–9/9; and control (CT) group, 16 lean cats with BCS 5/9.

The participating cats were recruited from the Veterinary Hospital clients through word-of-mouth, email communication, and posters. Obesity is a naturally occurring phenomenon that is not experimentally induced by diet. The exclusion criteria were cats with other endocrinopathies (diabetes mellitus), liver diseases, nephropathies, infectious diseases, pancreatitis, and neoplasia. The cats were considered healthy based on client histories, physical examination, and laboratory results. Laboratory analyses included complete blood counts, serum biochemistry profiles, and insulin concentrations.

Cats were classified as ideal (BCS of 5/9), OW (BCS of 6 or 7/9), or OB (BCS 8 or 9/9) based on a standard 9-point BCS system. Tape and digital scales were used to obtain morphometric body measurements and weigh each animal. A previously reported technique to assess body fat (BF) percentage was used to best ensure differences between study groups in the absence of dual-energy X-ray absorptiometry (DEXA) or magnetic resonance imaging. BF percentage was calculated using the following equation:

Percentage body fat (BF%) = ([(RC/0.7062) − LIM]/0.91560) − LIM

The RC was the circumference of the rib cage (cm), and LIM was the length of the lower limb from the middle of the patella to the dorsal hip (cm) [24].

Insulin resistance was estimated by calculating the homeostatic model assessment of insulin resistance (HOMA-IR) as follows [25–28]:

HOMA-IR = Glucose (mmol/L) × insulin (μU/mL)/22.5

### Blood sampling and processing

Five mL of blood were drawn from the jugular vein following a 12-h fasting. Blood was divided into 0.5 mL ethylenediaminetetraacetic acid tubes for a complete blood count and glucose measurement, 0.5 mL sodium fluoride tubes for glucose measurement, and 4 mL plain tubes for serum biochemistry profile and insulin concentration. The samples were centrifuged at 2000× *g* for 10 min after collection. The serum was stored at –80°C for future measurement of insulin, leptin, adiponectin, TNF-α, and IL-01 β.

Complete blood counts and serum biochemistry profiles were processed at the Veterinary Clinical Laboratory of the Veterinary Hospital. Serum biochemistry measurements were performed using the Cobas C111 Automatic Analyzer (Roche^®^ Diagnostics, Switzerland). Complete blood counts were performed using a Poch100iv-Diff (Roche^®^ Diagnostics, Switzerland) and light microscopy (Nikon^®^, Eclipse Ni-U, Japan). Insulin concentration was measured by chemiluminescence using a Cobas e411 analyzer (Roche^®^ Diagnostics, Switzerland) and a commercial Insulin Elecsys Cobas e 100.

### Measurement of inflammatory cytokines and adipokine

We analyzed the fasting serum levels of leptin, adiponectin, TNF-α, and IL-1β using enzyme-linked immunosorbent assay (ELISA) kits (Abcam PLC®, Cambridge, UK). The manufacturer’s protocol was followed for each sample in duplicate assays.

Leptin values (n = 51) were assessed with a human leptin assay ranging from 15.63–1000 pg/mL (ab179884, Abcam PLC^®^, Cambridge, UK). The intra-assay and inter-assay coefficients of variation were 5.1% and 3.5%, respectively; the assay sensitivity was 4.65 pg/mL, and the serum recovery averaged 91%, ranging from 90%–93%. The kit utilizes capture antibodies marked with an affinity tag that binds to the monoclonal antibody coating the plates. This approach to sandwich ELISA enables the simultaneous formation of antibody-analyte sandwich complexes.

Adiponectin (n = 51) was measured using a mouse adiponectin assay with a range of 1.5–6 ng/mL (ab108785, Abcam PLC^®^, Cambridge, UK). The intra-assay and inter-assay coefficient of variations (CVs) were 7.5% and 10.8%, respectively, the assay sensitivity was 0.23 ng/mL, and recovery was 100%. Adiponectin-specific antibodies were recoated onto 96-well plates and blocked. Test samples were added to the wells, and subsequently, an adiponectin-specific biotinylated detection antibody was added, _ followed by washing with wash buffer. Streptavidin-peroxidase conjugate was added, and unbound conjugates were washed away with a wash buffer. Tetramethylbenzidine (TMB) was then used to visualize streptavidin-peroxidase enzymatic reaction. TMB was catalyzed by streptavidin-peroxidase to produce a blue color product that changed to yellow after adding an acidic stop solution. The density of yellow coloration was directly proportional to the amount of adiponectin captured in the plate.

The human TNF-α assay (ab181421, Abcam PLC^®^) was used to measure TNF-α levels (n = 51) within a range of 15.63–1000 pg/mL. The CVs were 2.5% (intra) and 3.1% (inter-assay), sensitivity was 4.32 pg/mL, and recovery ranged from 83% to 93% with an average of 88%. The kit uses monoclonal antibodies for plate coating, which recognizes the affinity tag attached to capture antibodies. This approach to sandwich ELISA forms the antibody-analyte sandwich complexes in a single step.

IL-1β (n = 51) was measured using a human IL-1β assay with a range of 14.06–900 pg/mL (ab214025, Abcam PLC^®^). The intra-assay and inter-assay CVs were 4.8% and 5.6 %, respectively. The assay sensitivity was 5.64 pg/mL, and the recovery in serum samples was an average of 103% and ranged from 101 to 105%. This kit employs the same methodology as leptin and TNF-α explained above.

### Statistical analysis

The values obtained were organized in Microsoft Excel 2021 spreadsheet (Microsoft, Washington, USA), and data are presented as mean ± standard deviation. We used GraphPad Prism software version 8.0.1 for Windows (GraphPad, San Diego, California, USA) for statistical analyses and graphs. The distribution of continuous variables was assessed using the Shapiro–Wilk normality test. The one-way analysis of varience test was used to verify the statistical differences between leptin, adiponectin, TNF-alpha, IL-1, insulin, glucose, and HOMA-IR in the CT, OW, and OB groups, and the means were compared using the Tukey test at the 5% probability level. The relationship between weight, BF, and BMI was evaluated using the studied parameters with Pearson’s correlation coefficient. According to the value obtained by Pearson’s linear correlation coefficient (r), |0–0.2| was considered as follows: No correlation; |0.2–0.4|: Weak correlation; |0.4–0.7|: Moderate correlation; and |0.7–1.0|: Strong correlation. Next, linear regression was performed to identify the coefficient of determination (R²). Results with p < 0.05 were considered statistically significant.

## Results

Fifty-one cats were included in the study (males, n = 22; females, n = 29) and were neutered. The mean ± SD of age, weight, BCS, and BF% in the OB group was 4.50 ± 1.90 years, 6.09 ± 1.14 kg, 8.44 ± 0.51, and 43.51 ± 9.48%, respectively; the OW group was 4.11 ± 1.66 years, 5.09 ± 0.65 kg, 6.63 ± 0.50, and 31.69 ± 5.98%, respectively; and in the CG group 2.63 ± 1.82 years, 3.94 ± 0.72 kg, 5.00 ± 0.00, and 21.21 ± 5.25%, respectively. The three groups showed significant differences in weight, BCS, and BF% (p < 0.0001 in both). There were no differences between males and females with regard to age, weight, BCS, or BF%.

There were differences in mean values for some hematological parameters between groups. The OB group had a lower mean number of red blood cells (7.72 ± 0.32), higher mean corpuscular volume (48.51 ± 3.54), and higher total proteins (8.00 ± 0.52) compared with the OW (9.04 ± 1.27, 45.06 ± 4.30, and 7.63 ± 0.50) and CT group (8.95 ± 1.18, 44.48 ± 2.86; and 7.39 ± 0.49), respectively. The OW group had a lower mean number of total (9.70 ± 3.54) and segmented (6.15 ± 2.34) leukocytes than the CG (14.13 ± 5.94, 9.85 ± 5.86) and OB (11.54 ± 3.15, 8.15 ± 2.72) groups, respectively. No significant differences were observed for the other hematological variables evaluated.

Obesity (OB group) was associated with higher cholesterol (139.20 ± 42.88), triglyceride (136.00 ± 118.10), and urea levels (60.53 ± 7.77) compared with the OW (114.20 ± 35.98, 72.37 ± 27.75, and 57.87 ± 12.82) and CT group (84.94 ± 23.49, 48.50 ± 22.41, and 49.94 ± 6.10) groups, respectively. Higher cholesterol levels were also observed in the OW group compared with the CG group. There was no significant difference in glucose concentration between any groups. Biochemical changes remained within the reference interval for feline species, except for triglycerides and urea in the OB group, which showed higher values than the laboratory reference interval (Table-1).

**Table-1:** Changes in biochemical parameters (cholesterol, triglycerides, and glucose), insulin, and HOMA-IR between the CG, OW, and OB groups.

Parameters	CG (n= 16)	OW (n = 16)	OB (n =16)
Cholesterol	84.94 ± 23.49	114.20 ± 35.98**	139.20 ± 42.88**
Triglycerides	48.50 ± 22.41	72.37 ± 27.75	136.00 ± 118.10*
Glucose	117.30 ± 55.56	132.60 ± 42.36	125.30 ± 33.73
Insulin	2.84 ± 2.79	4.59 ± 2.33*	5.34 ± 3.50*
HOMA-IR	1.05 ± 1.67	1.56 ± 1.24*	1.91 ± 1.68*

Data are expressed as mean ± standard deviation (SD).

* Significant (p < 0.05) compared with the control group [one-way analysis of variance (ANOVA)].

** Significant (p < 0.01) compared with the control group (one-way ANOVA). HOMA-IR=Homeostatic model assessment of insulin resistance, CT=Control OW=Overweight, OB=Obese

In contrast to glucose, cats with OW 4.59 ± 2.33 and OB 5.34 ± 3.50 had significantly higher serum insulin concentrations than lean cats (CG group) 2.84 ± 2.79 viewed significant statistical differences (p = 0.008) between groups. Likewise, HOMA-IR index was higher in the OW (1.56 ± 1.24) and OB group (1.91 ± 1.68) groups than in the CG (1.05 ± 1.67) group (p = 0.0124 (Table-1).

**Figure-1:**
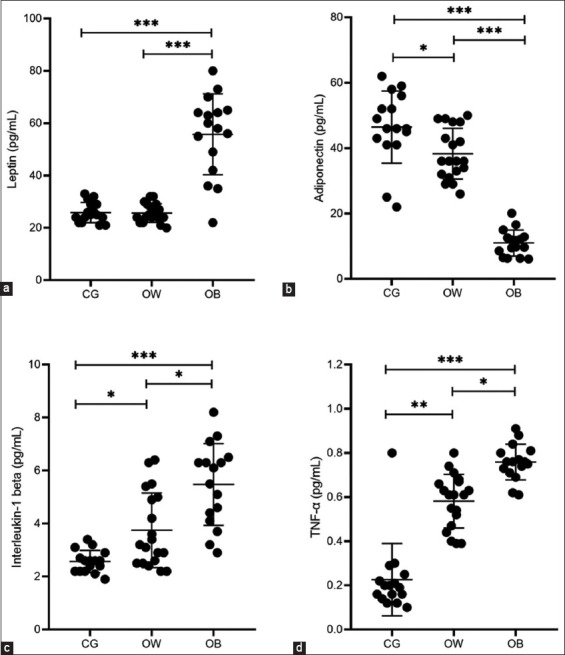
(a-d) Changes in leptin, adiponectin, interleukin-1β, and tumor necrosis factor-α concentrations between control overweight, and obese groups. *p < 0.05, **p < 0.01, ***p< 0.001.

OB cats demonstrated a significant increase in leptin (55.75 ± 15.45), TNF-α (0.76 ± 0.08) e IL-1β (5.48 ± 1.55) concentrations as compared with OW and lean cats (p < 0.0001). In contrast, adiponectin levels decreased in OB cats (11.00 ± 3.99) as compared with OW (38.32 ± 7.75) and lean cats (46.44 ± 11.04) with p < 0.0001. OW cats also exhibited a significant increase in the TNF-α (0.58 ± 0.12) and IL-1β (3.75 ± 1.55) levels compared with lean cats (p < 0.0001). However, leptin levels remained unchanged in OW cats (25.68 ± 3.53) compared to lean cats (25.88 ± 3.86) p= 0.99. As observed in the OB group, decreased adiponectin levels were also observed in the OW group (38.12 ± 7.75) compared with the CG group (46.44 ± 11.04). Leptin, adiponectin, and TNF-α e IL-1β concentrations are shown in Figure-1 for CT, OW, and OB cats.

**Figure-2:**
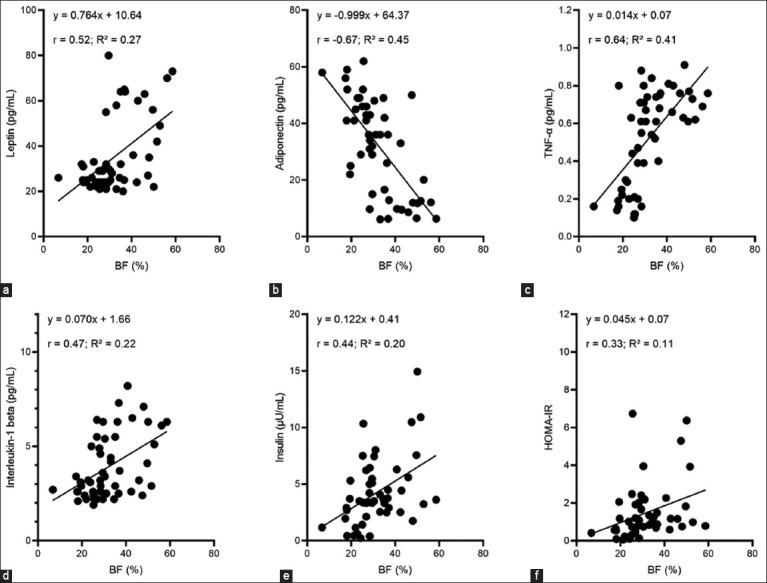
Correlation between body fat % and leptin pg/mL (a), adiponectin pg/mL (b), tumor necrosis factor-α pg/mL (c), interleukin-1β pg/mL (d), insulin μU/mL (e), and homeostatic model assessment of insulin resistance (f) variables.

Body weight showed a moderate correlation with leptin (r = 0.52), adiponectin (r = −0.67), and TNF-α (r = 0.64). Adiponectin decreased with increasing BF%, whereas leptin and TNF-α increased with BF%. Nevertheless, insulin (r = 0.44), HOMA-IR (r = 0.33), and IL-1β (r = 0.47) variables were moderately correlated with BF%. Weight (Wt) showed a moderate correlation with adiponectin (r = −0.55), TNF-α (r = 0.53), and IL-1β (r = 0.53), and a moderate correlation with leptin (r = 0.49) and insulin (r = 0.38) (Figure-2).

## Discussion

Clinical studies evaluating the biochemical, metabolic, and inflammatory profiles at different stages of obesity in client-owned cats are scarce, mainly in Brazil in the northeast region. In this study, we compared the circulating concentrations of glucose, triglycerides, cholesterol, insulin, leptin, adiponectin, TNF-α, and IL-1β in client-owned lean, OW, and OB cats treated routinely at the Veterinary Hospital of the State University of Ceará. Our results highlight several important findings. OB cats had important changes in their lipid profile, with a significant increase in triglycerides and total cholesterol, as well as changes in glucose metabolism with increased insulin and HOMA-IR index, leading to insulin resistance. Furthermore, there were changes in the adipokine secretion profile with increased leptin and diminished adiponectin levels and increased proinflammatory cytokines TNF-α and IL-1β concentration.

Obesity in cats is a major concern, as its prevalence has increased exponentially in recent decades [1]. Moreover, obesity has damaging health effects in cats [3, 5]. Similar to humans, several studies have reported that obesity in cats leads to peripheral insulin resistance and increased insulin concentrations [29–31]. Insulin resistance is a major factor in the pathogenesis of diabetes in cats, and relative insulin deficiency leads to hyperglycemia [31, 32]. Our study found elevated levels of insulin in OW and OB cats compared with lean cats; otherwise, glucose levels remained unchanged, as observed in other studies [29–31]. Early stages of insulin resistance can be reversible in OB cats who successfully lost weight, as evidenced by decreased insulin concentrations and the HOMA-IR index [33]. HOMA-IR is used to estimate insulin resistance and can assist in the early identification of insulin-resistant cats in clinical practice [26]. Our study evaluated HOMA-IR and measured fasting insulin and found that both variables were positively associated with BF%, suggesting that insulin resistance increases with increasing fat percentage. A similar result has been reported between HOMA-IR and OB cats [8, 27].

In OW cats, lipid metabolism often undergoes modifications. The literature and our study agree that OB and OW cats have higher serum total cholesterol levels and OB cats exhibit increased triglyceride levels. According to numerous studies [31, 34–36], OW and OB cats had significantly higher levels of triglycerides and total cholesterol in their routine biochemical profiles compared to lean cats. Insulin resistance in adipose tissue, caused by inflammation, obstructs insulin’s effect on lipolysis. In obesity, cytokines and endotoxin stimulate adipose tissue lipolysis. Therefore, mobilized fatty acids are esterified into triglycerides in the liver, and they may cause insulin resistance through failure of insulin signaling in liver tissue [37, 38]. The decrease in lipoprotein lipase activity may explain the lipid build-up observed in OB cats [39].

Obesity is a disorder characterized by chronic, low-grade systemic inflammation that results in dysregulation of adipokine and cytokine secretion [40], which may play a key role in the development of insulin resistance and diabetes mellitus type 2 and expanded risk of obesity-related cardiovascular disease, collectively referred to as metabolic syndrome in humans and other related species [19, 41, 42]. In cats, as in other species, leptin concentration increases with the expansion of fat mass and decreases with weight loss [21, 22, 43]. Nonetheless, the higher leptin concentrations in OB humans and cats do not appear to suppress appetite, leading to the mechanism of leptin resistance (involving reduced leptin signaling or decreased transport through the blood-brain barrier) in obesity [21]. The present study observed increased leptin and decreased adiponectin levels in OW and OB cats, as demonstrated in previous studies [22, 44, 45]. The concentrations of adiponectin and leptin are likely to change with changes in body condition and alter metabolism in OB cats. In our study, leptin was positively correlated with BF% and adiponectin was negatively correlated with BW. In other studies, leptin and adiponectin were positively and negatively correlated with insulin resistance in cats [22, 23, 29, 30]. The situation may be reversed during weight loss, as weight reduction induces a decrease in serum leptin and increases adiponectin, improving metabolic status [35, 46, 47].

Adipose tissue produces several other factors called proinflammatory cytokines, including TNF-α, interferon-γ (IFN-γ), and ILs, such as IL-1, IL-6, IL-8, and IL-10 [48, 49]. Circulating levels of these proinflammatory factors increase with the enlargement of fat mass. Many of these proinflammatory factors are produced by adipocytes and activated macrophages [50]. In the present study, we found a significant difference in the inflammatory markers TNF-α and IL-1β as compared to lean group with OW and OB groups; cats with OW and obesity had higher concentrations of these inflammatory markers. This suggests that obesity in cats also induces a chronic inflammatory response, as occurs in humans. The mechanisms involved are diverse and complex. An important characteristic of the inflammatory state caused by obesity may be related to the increased infiltration of macrophages, mast cells, and natural killer T cells in adipose tissue compared with lean tissue. This contributes to the increased expression of proinflammatory cytokines, metabolic pathophysiology, and generation of a chronic inflammatory state [51, 52]. These mechanisms may also occur in feline obesity.

In cats, few studies have linked obesity with an inflammatory profile. Hoenig *et al*. [29] and Hoenig *et al*. [30] reported that the concentrations of IL-1, IL-6, and TNF-α did not change in induced OB cats. They hypothesized that metabolism adapts more appropriately to a higher intake of calories during the initial phase of obesity. Furthermore, Zapata *et al*. [45] did not find significant differences in IL-12 concentrations between OW and diabetic cats. In contrast, another study identified accumulations of IL-1β and IL-6 in the pancreatic islets of OB and hyperglycemic cats [53]. Other studies suggest that obesity could predispose individuals to oxidative stress by damaging mitochondrial function through morphological alterations, increased membrane peroxidation and reactive oxygen species (ROS), decreased ATP levels, and abnormal mitochondrial fatty acid β-oxidation in liver [54, 55]. Many differences in secretion of TNF-α by adipose tissue occur among species. In mice, TNF-α is released into the systemic circulation [19]. Circulation patterns of adipose and blood-derived TNF-α are not clearly defined in cats. In OB cats, both the concentration and mRNA expression of TNF-α were higher in adipose tissue [44, 56]. The concentration of IL-1β is increased in OB humans [57], with the combination of elevated IL-1β and IL-6 increasing the risk of both type II diabetes and metabolic syndrome [58]. High concentrations of IL-1β were observed in inflammatory conditions such as *Cytauxzoon felis* infection, mesenteric ischemia, and sepsis [59, 60] in cats instead of obesity. Our study is the first to detect higher concentrations of IL-1β in spontaneous and chronic OW and OB cats.

## Conclusion

Our study used client-owned cats as the study population; thus, many variables may be involved in the development of obesity in these cats. Our results may differ from those of other studies due to the use of naturally OB cats instead of experimentally induced obesity in cats.

This study is the first to demonstrate a higher serum concentration of inflammatory markers in client-owned OW and OB cats. The preliminary results showed increased levels of proinflammatory markers in OW cats, suggesting obesity-induced inflammation in cats. More studies are needed to understand the pathways through obesity that cause inflammation in OW and OB cats.

## Authors’ Contributions

SLA: Conceptualization, methodology, investigation, data curation, and drafted the manuscript. PML: Investigation, data curation, and reviewed and edited the manuscript. THSP: Software and formal analysis. TLS and RRPPBM: Validation and resources. MDRC and AMCM: Validation and investigation. INGS: Methodology and resources. GBM and JSAME: Conceptualization, supervision, and reviewed and edited the manuscript. All authors have read, reviewed, and approved the final manuscript.
